# Arthroscopic-Assisted Lower Trapezius Tendon Transfer With Autologous Semitendinosus Tendon and Long Head of Biceps Superior Capsule Reconstruction for Massive Irreparable Posterosuperior Rotator Cuff Tears

**DOI:** 10.1016/j.eats.2022.03.005

**Published:** 2022-06-21

**Authors:** Chih-Hao Chiu, Cheng-Pang Yang, Hao-Che Tang, Chun-Jui Weng, Kuo-Yao Hsu, Alvin Chao-Yu Chen, Yi-Sheng Chan

**Affiliations:** a Department of Orthopedic Surgery, Chang Gung Memorial Hospital, Taoyuan, Taiwan; b Bone and Joint Research Center, Chang Gung Memorial Hospital, Linkou, Taiwan; c Comprehensive Sports Medicine Center, Chang Gung Memorial Hospital, Taoyuan, Taiwan; d Department of Orthopedic Surgery, Chang Gung Memorial Hospital, Linkou, Taiwan; e Department of Orthopedic Surgery, Chang Gung Memorial Hospital, Keelung, Taiwan

## Abstract

We present a surgical technique combining arthroscopic-assisted lower trapezius tendon (LTT) transfer with autologous semitendinosus tendon and long head of biceps tendon (LHBT) superior capsule reconstruction (SCR) for massive irreparable posterosuperior rotator cuff tears. The patients are placed in the beach-chair position with the ipsilateral lower leg prepared simultaneously. After both tendons are harvested, 1 limb of a semitendinosus graft is fixed with the LTT via a Krakow suture. The LHBT is then fixed by an anchor 5 to 8 mm posterior to the bicipital groove and tenotomized distally. The transverse humeral ligament is released afterward to provide better visualization. A Beath pin is introduced from anterolateral portal, aiming at the bicipital groove, and drilled posteriorly until it exits at the infraspinatus footprint. Next, 4.5- and 8-mm cannulated drills are used sequentially to create a humeral tunnel. A shuttle suture passed through infraspinatus fascia in the back brings the EndoButton and looped semitendinosus graft from posterior to anterior of the humerus, until the EndoButton flips and is fixed inside the bicipital groove. The shoulder is placed in 45° abduction and 30° external rotation. The free limb of semitendinosus tendon is then sutured with LTT with the desired tension.

Plenty of surgical techniques have been developed to treat massive irreparable posterosuperior rotator cuff tear, including simple debridement,^1^ margin convergence,^2^^,^^3^ biceps graft,^4^, ^5^, ^6^ partial repair,^7^^,^^8^ medialized footprint,^9^ superior capsule reconstruction (SCR) with autologous iliotibial band (ITB), or long head of the biceps (LHBT),^10^, ^11^, ^12^, ^13^ latissimus dorsi transfer (LDT),^14^ and lower trapezius tendon (LTT) transfers with Achilles allograft^15^^,^^16^ or hamstring autograft.^17^ Among them, LHBT SCR has gained popularity recently because it is available locally, free of additional costs, less technically demanding, and normalizes superior migration and subacromial contact pressure because of the spacer effect provided by the LHBT.^18^ Also, LTT transfer provides both greater excursion and a vector more similar to infraspinatus and teres minor compared to the LDT, resulting in an improved anteroposterior balancing force across the glenohumeral joint.^19^^,^^20^ Both autologous and allograft hamstring tendon and Achilles tendon allograft have been described in the literature to be augmented with LTT.^21^ In this article, we present a surgical technique combining arthroscopic-assisted LTT with autologous semitendinosus tendon and LHBT SCR for massive irreparable posterosuperior rotator cuff tears. This technique aims to stabilize the humeral head superiorly and posteriorly at the same time.

## Operative Technique

### Surgical Indications

The indications and contraindications of arthroscopic-assisted LTT with autologous hamstring tendon and LHBT SCR for massive irreparable posterosuperior rotator cuff tears are listed in Table 1.

**Table. 1:** Indications and Contraindications

Indications
Lack of active external rotation with the arm at the side, a hornblower sign, limitation in active abduction and forward elevation
Irreparable posterosuperior massive rotator cuff tears with Hamada stage ≤2
MRI demonstrating a massive irreparable tear of the posterosuperior rotator cuff
MRI demonstrating fatty infiltration of the infraspinatus muscle (grade >2 Goutallier classification)
Failed conservative treatment
Existing LHBT
Contraindications
Active forward elevation of ≤80° with an anterosuperior escape of the humeral head
Associated subscapularis tear (grade >II Lafosse classification)
Significant glenoid or humerus bone defects
Glenohumeral arthritis
Absent LHBT
Shoulder stiffness
Deltoid palsy

Abbreviations: LHBT, long head of the biceps; MRI, magnetic resonance imaging.

### Patient Preparation and Arthroscopic Portals

**Fig 1:**
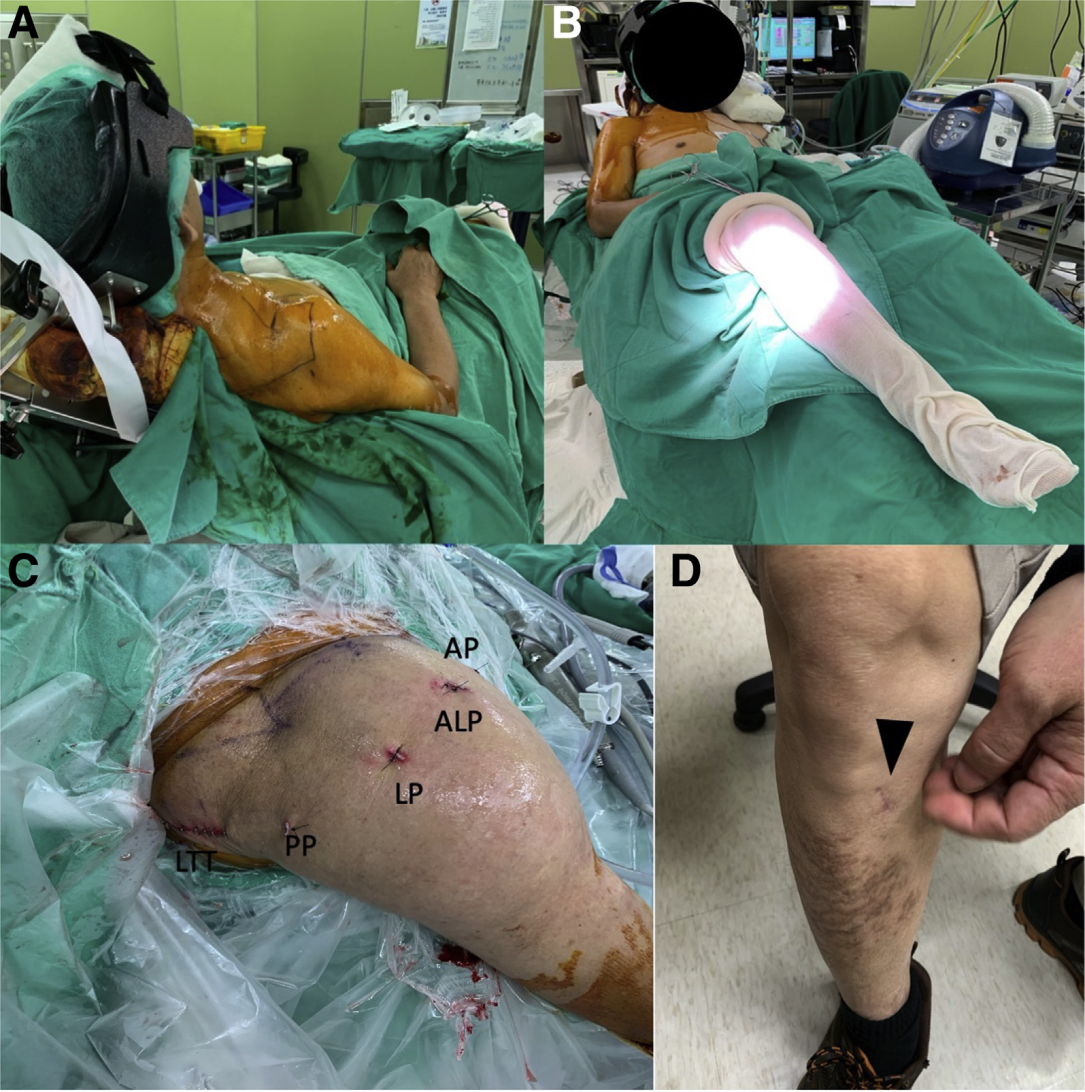
Patient position and arthroscopic portals, right shoulder, beach chair position. (A) All patients had general anesthesia with interscalene nerve block and were placed in the beach-chair position with a traction device. (B) The ipsilateral lower leg is prepared simultaneously. (C) Normally 3 arthroscopic portals are needed. (D). The semitendinosus tendon harvest site (arrowhead, the insertion of pes anserinus), right lower leg. Abbreviations: ALP, anterolateral portal; AP, anterior portal; LP, lateral portal; LTT, lower trapezius tendon; PP, posterior portal.

All patients have general anesthesia with interscalene nerve block and are placed in the beach-chair position with a traction device (Fig 1A). The ipsilateral lower leg is prepared simultaneously (Fig 1B). Usually, 3 arthroscopic portals are necessary: posterior, lateral, and anterolateral. If the subscapularis is torn and needs to be repaired, an additional anterior portal will be made. The medial border of the scapula, the scapula spine, and the tendon insertion of the LTT are marked on the skin (Fig 1C). The semitendinosus autograft is harvested from the insertion of pes anserinus (Fig 1D).

### Surgical Technique

#### Harvest and Preparation of Lower Trapezius Tendon

**Fig 2:**
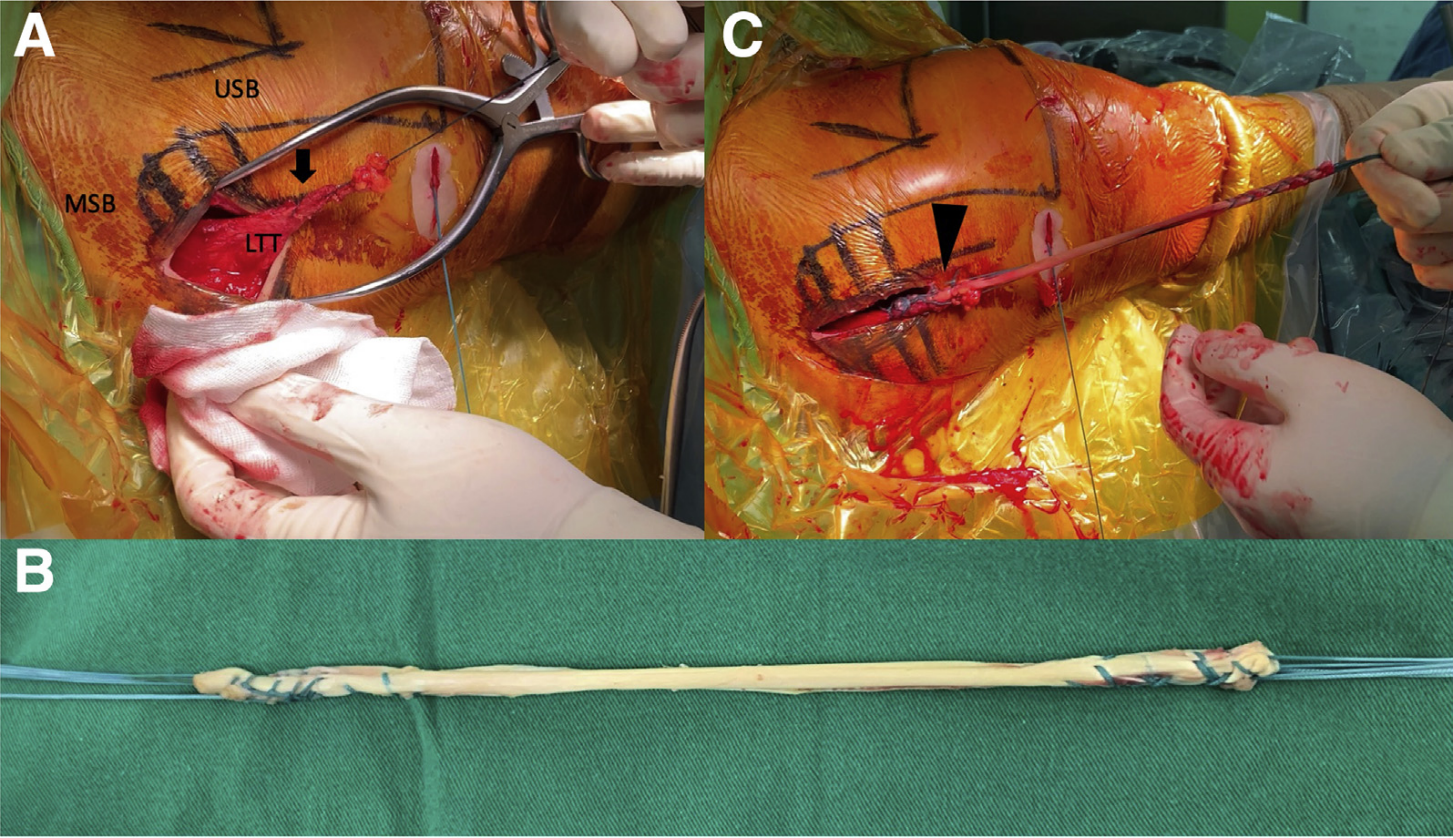
Harvest and preparation of lower trapezius tendon and semitendinosus tendon, right shoulder. (A) An 8-cm horizontal incision is made just below the spine of the scapula over the lower trapezius tendon insertion. The tendon part of LT was whipstitched with no. 2 Ethibond (arrow) to facilitate further manipulation. (B) The semitendinosus autograft was harvested with both ends sutured with no. 2 Ethibond. (C) One limb of semitendinosus graft was fixed with the tendon part of harvested LTT via a Krackow technique (arrowhead). Abbreviations: LTT, lower trapezius tendon; MSB, medial scapular border; USB, upper scapular border.

An 8-cm horizontal incision is made just below the spine of the scapula over the lower trapezius tendon insertion. The subcutaneous tissue is dissected, and the underlying fat is removed until the tendon is identified. The LTT is then detached from the scapula spine and mobilized superiorly from the middle trapezius and medially until the medial border of scapula. Care must be taken to avoid injury to the spinal accessory nerve that runs 3 to 4 cm medial to the scapula. The tendon part of LT was whipstitched with no. 2 Ethibond (Ethicon) to facilitate further manipulation (Fig 2A).

#### Harvest and Preparation of Semitendinosus Tendon with Lower Trapezius Tendon

The semitendinosus autograft was harvested full length from the insertion site with a tendon stripper (Smith & Nephew Endoscopy, Andover, MA) with both ends sutured with no. 2 Ethibond (Fig 2B). One limb of semitendinosus graft was fixed with the tendon part of harvested LTT via a Krackow technique (Fig 2C).

#### Superior Capsule Reconstruction With Long Head of Biceps Tendon

**Fig 3:**
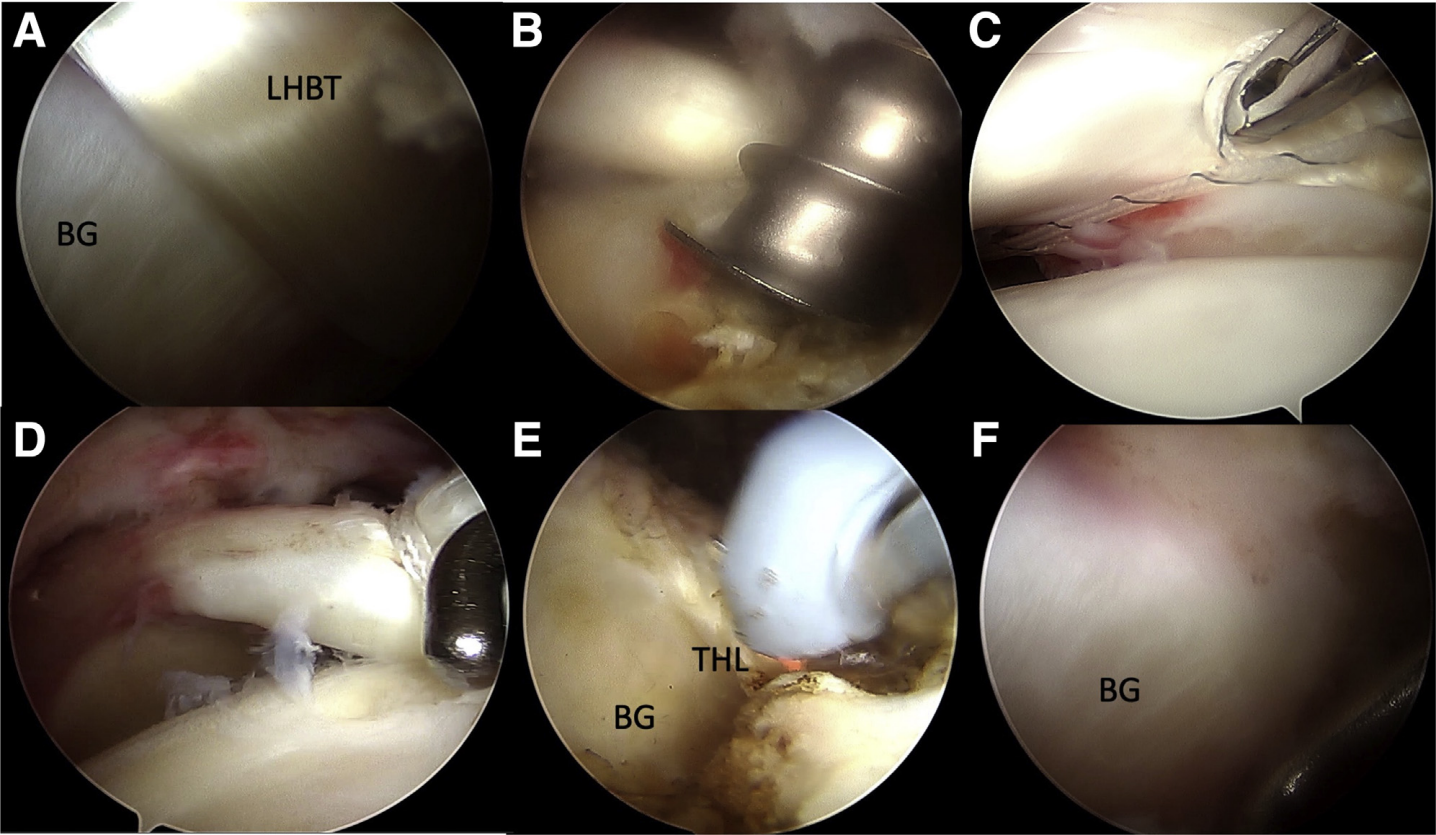
Superior capsule reconstruction with long head of biceps tendon, right shoulder, viewed from lateral portal. (A) The bicipital groove and LHGT are visualized. (B) A suture-based anchor is passed from anterolateral portal and inserted 5-8 mm posterior to the bicipital groove near the cartilage of humerus. (C) One lasso-loop is made by a suture manipulator and CleverHook. (D) The lateral part of the LHBT is rerouted posteriorly, proving a strong spacer effect. (E) THL is released. (F) The bicipital groove is cleared after LHBT tenotomy and THL release, providing better visualization for humeral tunnel drilling and graft passage. Abbreviations: BG, bicipital groove; LHBT, long head of the biceps tendon; THL, transverse humeral ligament.

Viewing from lateral portal, a suture-based anchor is passed from anterolateral portal and inserted 5-8 mm posterior to the bicipital groove near the cartilage of humerus. The surgical techniques are the same as Chiu et al.^22^ described previously (Fig 3A-F). We release the transverse humeral ligament once the LHBT is rerouted and fixed posteriorly. This will provide better visualization for humeral tunnel drilling and graft passage.

#### Humeral Tunnel Drilling and Graft Passage

**Fig 4:**
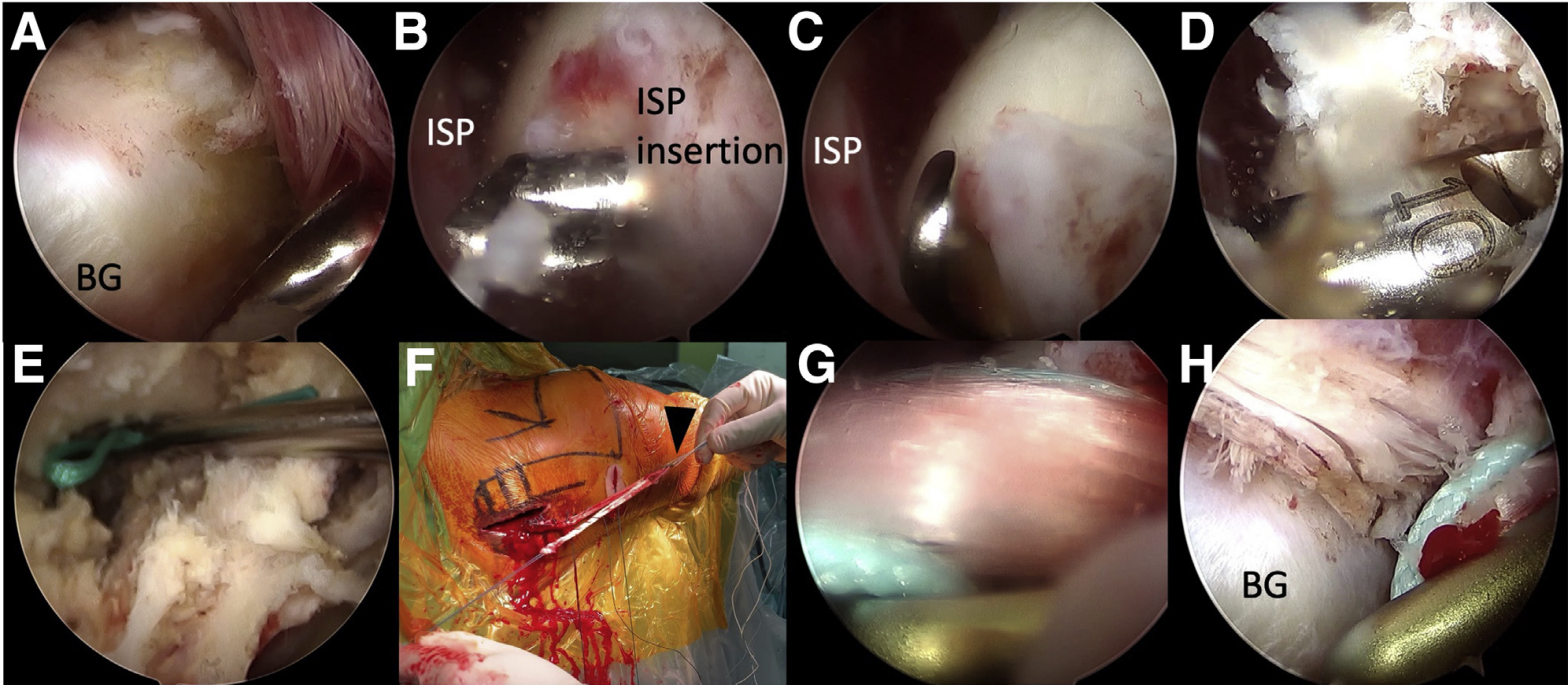
Humeral tunnel drilling and graft passage, right shoulder, viewed from lateral portal. (A) A Beath pin is introduced from anterolateral portal, aiming at bicipital groove. (B) The pin is drilled posteriorly until it exits at the upper part of native infraspinatus tendon insertion point. (C) A 4.5-mm rigid cannulated drill is first used to ream from anterior to posterior to create a humeral tunnel. The length of the tunnel is measured. (D) An 8-mm rigid cannulated drill is used to ream from posterior to anterior until the desired length inside the humeral tunnel. (E) A grasper is inserted along the length of the infraspinatus muscle, and the shuttling suture is pulled out of the opening of the infraspinatus fascia. (F) The free limb of semitendinosus tendon not fixed with LTT is passed from the loop of a 20-mm EndoButton (arrowhead) and works in a double fashion. (G and H) The EndoButton is passed intra-articularly from posterior to anterior, until it exits the bicipital groove. Abbreviations: BG, bicipital groove; ISP, intraspinatus; LTT, lower trapezius tendon.

Viewing from lateral portal, the bicipital groove is identified. A Beath pin (Smith & Nephew Endoscopy) is introduced from anterolateral portal, aiming at bicipital groove (Fig 4A), which is the hardest bone on the anterior aspect of the humeral head, and drilled posteriorly until the pin exits at the upper part of native infraspinatus tendon insertion point (Fig 4B). A 4.5-mm rigid cannulated drill is first used to ream from anterior to posterior to create a humeral tunnel. The length of the tunnel is measured (Fig 4C). After introducing the Beath pin into the humeral tunnel again, we use an 8-mm rigid cannulated drill to ream from posterior to anterior until the desired length inside the humeral tunnel (Fig 4D). A suture shuttle is then passed from posterior to anterior and retrieved out of the anterolateral portal. The infraspinatus fascia is then opened, facilitating suture and graft passage. A grasper is then inserted along the length of the infraspinatus muscle, and the shuttling suture is pulled out of the opening of the infraspinatus fascia (Fig 4E), out of the wound of LTT harvest. The free limb of semitendinosus tendon not fixed with LTT is passed from the loop of a 20-mm EndoButton CL (Smith & Nephew Endoscopy) and works in a double fashion (Fig 4F). The leading and flipping sutures of the EndoButton are tied with the shuttling suture and passed intra-articularly from posterior to anterior (Fig 4G), until it exits the bicipital groove (Fig 4H).

#### Tensioning of Lower Trapezius Tendon and Semitendinosus Graft

**Fig 5:**
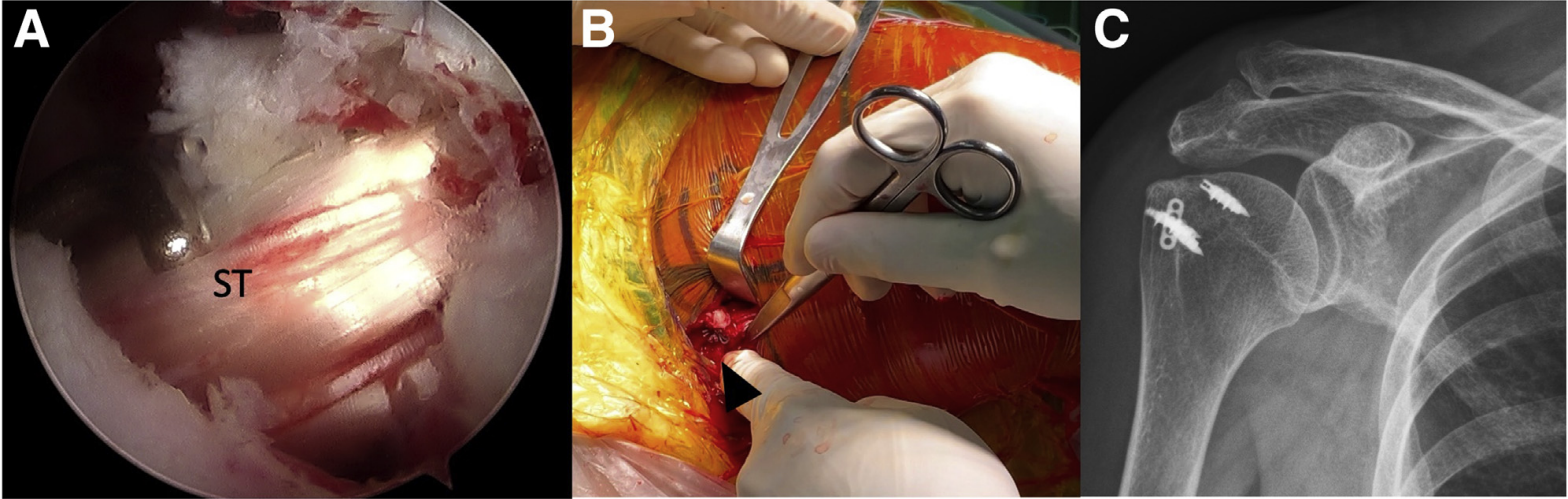
Tensioning of lower trapezius tendon and semitendinosus graft, right shoulder. (A) After the EndoButton is flipped and fixed at the bicipital groove, the tension of the ST graft is checked intra-articularly under arthroscopy. (B) The other limb of ST graft is fixed side by side with the LTT with Krakow suture (arrow). (C) Postoperative x-ray revealing EndoButton fixed inside bicipital groove. Abbreviations: LTT, lower trapezius tendon; ST, semitendinosus.

After the EndoButton is flipped and fixed at the bicipital groove, the shoulder is placed in 45° abduction and 30° external rotation. The free limb of semitendinosus tendon not yet fixed with LTT can be pulled backward to the desired tension. After adequate tension is achieved and checked intra-articularly under arthroscopy (Fig 5A), this end can be fixed side by side with the LTT with Krakow suture (Fig 5B). The patient is placed in a brace set at 45° abduction and 30° external rotation to relieve tension on the reconstruction after final fixation. Postoperative x-ray is shown in Fig 5C.

### Postoperative Protocol

The shoulder is protected in the brace for the first 6 weeks. From 6 to 12 weeks, active range of motion is permitted, with avoidance of any activation of the LTT transfer. Three months after the operation, the patients are allowed to perform shoulder abduction and external rotation with scapular retraction.

**Fig 6:**
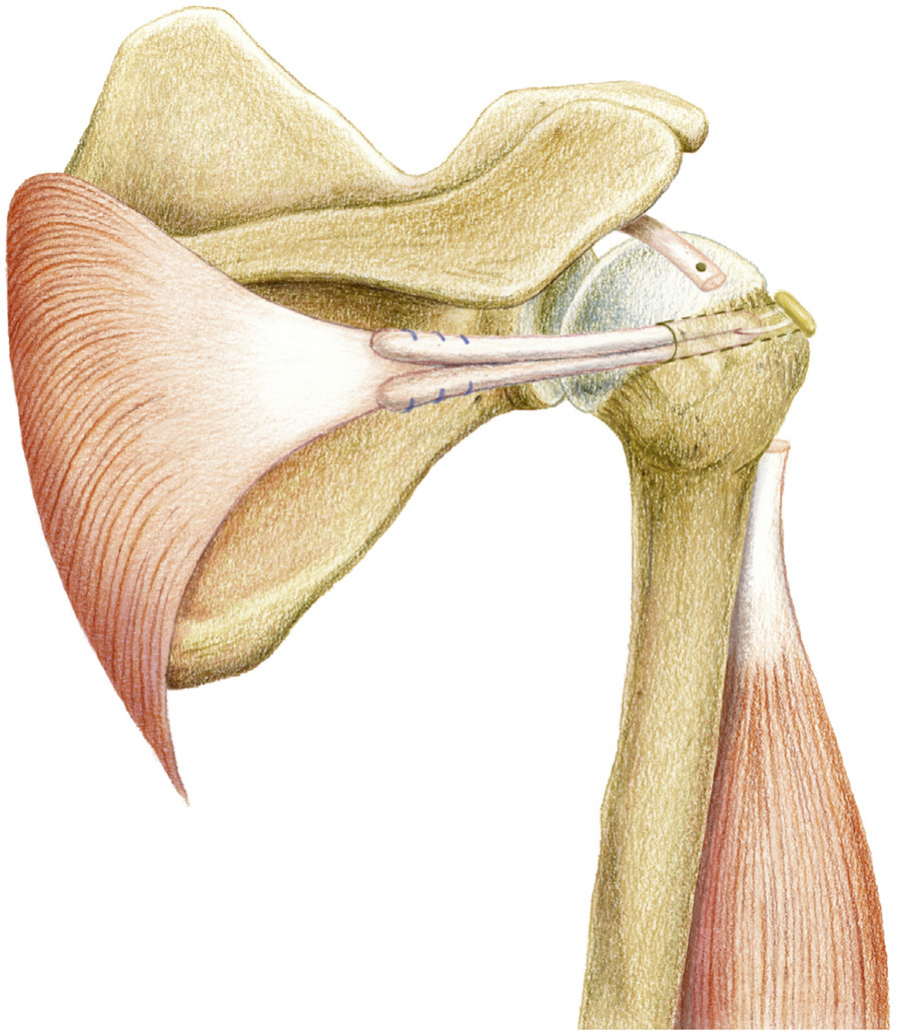
The final construct of the reconstruction.

The whole procedure of the surgery is shown in the Video 1. The pearls/pitfalls of the surgical steps are shown in Table 2. The advantages, risks, and limitations of the technique are shown in Table 3. The final construct is shown in Fig 6.

**Table 2:** Surgical Steps, Tips, Pearls, and Pitfalls

Surgical Step	Tips and Pearls	Pitfalls
Patient preparation and arthroscopic portals	1. Three arthroscopic portals: posterior, lateral, anterolateral	Normally the cannula is not needed.
	2. An additional anterior portal is needed for subscapularis repair.	
	3. The ipsilateral lower leg is prepared simultaneously.	
Harvest and preparation of lower trapezius tendon	1. An 8-cm horizontal incision is made just below the spine of the scapula over the lower trapezius tendon insertion.	Care must be taken to avoid injury to the spinal accessory nerve that runs 3 to 4 cm medial to the scapula.
	2. The LTT is detached from the scapula spine and mobilized superiorly from the middle trapezius and medially until the medial border of scapula.	
	3. The tendon part of LT is whipstitched with no. 2 Ethibond.	
Harvest and preparation of semitendinosus tendon with lower trapezius tendon	1. The semitendinosus autograft is harvested full length from the insertion site with a tendon stripper.	
	2. Both ends are sutured with no. 2 Ethibond.	
	3. One limb of semitendinosus graft is fixed with the tendon part of harvested LTT via a Krackow technique.	
Superior capsule reconstruction with long head of biceps tendon	1. Viewing from lateral portal, a suture-based anchor is passed from anterolateral portal and inserted 5-8 mm posterior to the bicipital groove near the cartilage of humerus.	The proximal attachment of the LHBT on the glenoid should be preserved to avoid an unstable biceps root.
	2. One lasso-loop is made by a suture manipulator and CleverHook.	Be careful not to cut the suture during LHBT tenotomy and THL release.
	3. The radiofrequency cautery device is used to tenotomize the LHBT at the entrance of the bicipital groove.	
	4. Tension of the LHBT can be made by penetrating the intra-articular LHBT in a more medial position by the 2nd and 3rd lasso-loop.	
	5. The proximal attachment of the biceps on the glenoid side is preserved, providing native fixation.	
	6. The lateral part of the LHBT is rerouted posteriorly, providing a strong spacer effect.	
	7. The THL is released once the LHBT is rerouted and fixed posteriorly, providing better visualization for humeral tunnel drilling and graft passage.	
Humeral tunnel drilling and graft passage	1. A Beath pin is introduced from anterolateral portal, aiming at the bicipital groove.	The Beath pin should be put low enough in the bicipital groove to avoid intra-operative humeral fracture during tunnel preparation.
	2. The Beath pin is drilled posteriorly until it exits at the upper part of the native infraspinatus tendon insertion point.	
	3. A 4.5-mm rigid cannulated drill is first used to ream from anterior to posterior to create a humeral tunnel.	
	4. The length of the tunnel is measured.	
	5. An 8-mm rigid cannulated drill reams from posterior to anterior until the humeral tunnel is the desired length.	
	6. A suture shuttle is passed from posterior to anterior and retrieved out of the anterolateral portal.	
	7. A grasper is inserted along the length of the infraspinatus muscle, and the shuttling suture is pulled out of the opening of the infraspinatus fascia.	
	8. The free limb of semitendinosus tendon not fixed with LTT is passed from the loop of a 20-mm EndoButton and works in a double fashion.	
	9. The leading and flipping sutures of the EndoButton are tied with the shuttling suture and passed intra-articularly from posterior to anterior, until it exits the bicipital groove.	
Tensioning of lower trapezius tendon and semitendinosus graft	1. After the EndoButton is flipped and fixed at the bicipital groove, the shoulder is placed in 45° abduction and 30° external rotation.	In osteoporotic patients, the semitendinosus should be tensioned gradually.
	2. The free limb of semitendinosus tendon is pulled backward until the desired tension checked intra-articularly.	
	3. This end is fixed side by side with the LTT with a Krakow suture.	

Abbreviations: LHBT, long head of the biceps; LTT, lower trapezius transfers; THL, transverse humeral ligament.

**Table 3:** Advantages, Risks, and Limitations

Advantages
1. Easier to harvest LTT than LDT.
2. No need to harvest ITB for SCR.
3. Treat biceps lesion simultaneously with LHBT SCR.
4. Provide a better spacer effect by LHBT SCR than LTT alone.
5. More anatomic reconstruction of the anterior rotator cable with LHBT SCR.
6. LTT provides better biomechanical properties than LDT.
7. Easier humeral tunnel drilling from anterior to posterior after LHBT SCR because of the clear bicipital groove.
8. EndoButton provides strong fixation and versatility to adjust the final tension.
9. Autologous semitendinosus incorporates faster and reduces the risk of inflammatory response versus Achilles tendon allograft for LTT.
Risks
1. Humeral fracture during tunnel preparation if the drill is put too high in the bicipital groove.
2. Popeye deformity of the forearm after biceps tenotomy.
3. Biceps tenotomy is associated with cosmetic deformity, cramping, and weakness.
4. Elongation of the biceps muscle-tendon unit after rerouting may happen if biceps tenotomy is not done, which potentially leads to an increase in the tension and anchor pullout.
Limitations
1. No full reconstruction of the supraspinatus footprint than LTT with Achilles tendon allograft.
2. Possible degenerated biceps tendon.
3. Extensive arthroscopic technique.
4. Further clinical and radiological follow-up should be done.

Abbreviations: ITB, iliotibial band; LDT, latissimus dorsi transfer; LHBT, long head of the biceps; LTT, lower trapezius transfers; SCR, superior capsule reconstruction.

## Discussion

Open LTT transfer has been used to restore external rotation in patients with brachial plexus injuries, with promising results.^23^^,^^24^ In 2016, Elhassan et al.^15^ described the technique of arthroscopic-assisted LTT transfer as an alternative to LDT transfer^14^ for irreparable posterosuperior cuff tear, and it gained popularity. Although LDT has a greater excursion than trapezius tendon,^25^ it is biomechanically less favorable regarding the anteroposterior balancing force and the compressive forces than LDT.^20^ LTT transfer also produced values similar to an intact cuff during external rotation in abduction.^21^ Both autologous hamstring tendon^26^ and Achilles tendon allograft^16^ have been used for LTT with onlay or inlay fixation methods.^21^ In our technique, we used autologous semitendinosus tendon as a graft to incorporate faster and reduce the risk of inflammatory response. We also tubularized the tendon into a loop fashion to increase the diameter of the graft through the transosseous tunnel and fixed it with an EndoButton, which is believed to be the strongest fixation in vitro.^27^ One limb of semitendinosus graft was first fixed at harvested LTT. The other limb was used to adjust the final tension by pulling the free limb, as a tension of 24 N is the most effective at restoring initial vectors on the humeral head and the scapula.^20^

Regarding the humerus tunnel drilling during the surgery, Valenti and Werthel^26^ used a guiding device to create a bone tunnel from posterior of the infraspinatus footprint to the bicipital groove anteriorly, which the authors considered difficult because the guide might be too bulky. Ek et al.^28^ applied a guidewire passed from anterior to posterior, with the goal being that the pin exits at the upper part of native infraspinatus tendon insertion point, which might be a better solution to facilitate the surgery. We tenotomized the LHBT distally as Boutsiadis et al.^11^ proposed after LHBT SCR, which made drilling from the anterior to posterior part of the humerus easier because the whole bicipital groove was cleared after the proximal part of LHBT been rerouted posteriorly onto the footprint of supraspinatus. In this way, the EndoButton can sit tight inside the bicipital groove without motion. A regular cannulated drill for cruciate ligament reconstruction can be used.

The anterior rotator cable is the primary force-transmitting structure at the proximal humerus.^29^ Therefore, we fixed the LHBT as an SCR 5-8 mm posterior to the bicipital groove near the cartilage of the humerus to provide an anatomic reconstruction of anterior cable. It is locally available than ITB, providing a static supporting structure to help maintain glenohumeral congruency, and acts to prevent humeral head superior migration.^18^ Barth et al.^10^ have proved that the LHBT SCR provided a significantly better infraspinatus tendon healing rate than conventional double-row group and transosseous equivalent with patch augmentation group on 24-month ultrasound follow-up.

## Supplementary Data

Full ICMJE author disclosure forms

Video 1 The whole procedure of the surgery. This is a 60-year-old male heavy worker who worked at a construction area with preoperative pseudoparesis. Active forward elevation was about 80°. He also had external rotation lag and positive Hornblower sign. Active internal rotation was limited to the sacrum. No osteoarthritis was seen on the plain x-ray, but a massive posterosuperior cuff tear with retraction to glenoid level was observed. Both supraspinatus and infraspinatus had Goutallier 3 fatty infiltration. The patient was put in a beach-chair position with ipsilateral knee prepared as well. The semitendinosus tendon was harvested with both ends sutured with no. 2 Ethibond. The LTT was detached, and the tendon part of it was whipstitched to facilitate further manipulation. Viewing from lateral portal, a basic arthroscopy exam was performed to confirm the retractability of the cuff. A suture-based anchor is passed from anterolateral portal and inserted 5-mm posterior to the bicipital groove near the cartilage of the humerus. One loop was passed through the biceps first, and then we used a bird beak to penetrate the biceps. After knot tying, the biceps was rerouted posteriorly. Then we cut the biceps distal to our knots at the entrance of the bicipital groove. The proximal attachment of the biceps on the glenoid side was preserved, providing native fixation, while the lateral part worked as a LHBT SCR. Then the THL was released, providing better visualization for humeral tunnel drilling and graft passage. More tension could be given by penetrating the intra-articular LHBT in a more medial position. A strong spacer effect was achieved. Then we use a Beath pin introduced from anterolateral portal, aiming at the bicipital groove, and drilled posteriorly until it exited at the upper part of the native infraspinatus insertion point. A 4.5-mm cannulated drill was used to ream from anterior to posterior to create a humeral tunnel, and the length of the tunnel was measured. Another 8-mm drill was used to ream from posterior to anterior until the desired length. Then we passed a suture along the infraspinatus muscle, out of the opening of the infraspinatus fascia. The free limb of semitendinosus tendon not fixed with LTT was passed from the loop of a 20-mm EndoButton and worked in a double fashion. The EndoButton was shuttled intra-articularly from posteriorly to anteriorly with the graft until it exited the bicipital groove and flipped for final fixation. The free limb of semitendinosus tendon was then pulled backward until the desired tension, checked intra-articularly. This end was fixed side by side with the LTT with Krakow suture. Additional sutures could be made onto the remnant cuff, providing more soft tissue coverage on top of the humeral head. Although it might be a nonfunctional cuff, we hoped it could provide some additional strength to stabilize the humerus. Postoperative plain x-ray showed better acromiohumeral distance right after the surgery. Six months after the surgery, the patient had about 130° of active forward elevation and acceptable active external rotation without lag sign. The acromiohumeral distance was preserved. The final construct was like this cartoon.
